# Meeting report: the Human Genome Meeting (HGM) 2019 in Seoul, Korea

**DOI:** 10.1186/s40246-019-0218-2

**Published:** 2019-08-01

**Authors:** Angela Solano, Giuseppe Novelli, Shruti Baghat, Piero Carnici, Gert-Jan van Ommen, Juergen K. V. Reichardt

**Affiliations:** 1 INBIOMED, Facultad de Medicina, UBA-CONICET and Genotipificacion, DAC, CEMIC, Buenos Aires, Argentina; 2 Department of Biomedicine and Prevention, Tor Vergata University of Rome, Rome, Italy; 3 RIKEN Center for Integrative Medical Sciences, Yokohama, Japan; 4 Department of Human Genetics, Leiden University Medical Center (LUMC), Leiden, The Netherlands; 5 Australian Institute of Tropical Health and Medicine (AITHM), James Cook University, Smithfield, QLD 4878 Australia; 6 Neuromed, IRCCS, Pozzilli, Isernia Italy

**Keywords:** Human genomics, Sequencing, Ethics, Microbiome

The 2019 Human Genome Meeting (HGM2019) of the Human Genome Organization (HUGO) was held in Seoul, Korea, from 24 to 26 April 2019, at Ewha Woman’s University on their beautiful campus (https://en.wikipedia.org/wiki/Ewha_Womans_University). The twenty-third HGM was a truly international event, attracting some 507 registrants from 29 countries spanning 6 continents who interacted both formally and informally at 4 plenary lectures, 10 parallel scientific sessions, 10 ancillary events, such as workshops, luncheon sessions, 2 social events, poster sessions, and interactions with exhibitors. (https://www.hugo-hgm.org/program/scientific). Furthermore, the HGM in Seoul featured 42 international and 7 local distinguished speakers highlighting the worldwide draw of this annual meeting.

## Selected highlights from the plenary sessions

Diana Bianchi (National Institutes of Health, NIH, USA) presented in her lecture entitled “Prenatal Genomic Medicine: Transforming Obstetrical Practice and New Biological insights” her view on the biggest example of the impact of genomics in clinical medicine. The presentation was based on the description of the changes in obstetric practice as a result of the application of non-invasive prenatal testing (NIPT). The reduction of invasive diagnostic procedures is an important and relevant goal since miscarriages as a consequence of older procedures would be eliminated. It is important though that any false-positive results (a very low percentage, indeed) may be a probable finding, to be verified with other methodologies before arriving at a final conclusion and making clinical decisions.

Harry Ostrer (Albert Einstein College of Medicine, USA) presented his personal journey through human genetics and genomics as a physician and researcher over some 30+ years. Harry’s talk entitled “Personalized Medicine, Precision Medicine and Global Health” highlighted the scientific and life insights he gathered over the decades which include his ethnic group’s genetic history and 103+ prevalent mutations that could serve as group-specific preventive genetics in (Ashkenazi) Jews. He highlighted the importance of dissecting genetic mechanisms and pathways using contemporary technologies for understanding the pathogenesis of disorders of sex development and other disorders. He showed how lessons from rare diseases of DNA repair could be applied for understanding pathways of common cancer susceptibilities and that challenging oneself to move beyond one’s comfort zone could lead to more impactful research. Ostrer also discussed his role as a sole plaintiff with standing in the landmark BRCA case against Myriad Genetics in the USA, which reset the framework for intellectual property in human genetics.

**Fig. 1:**
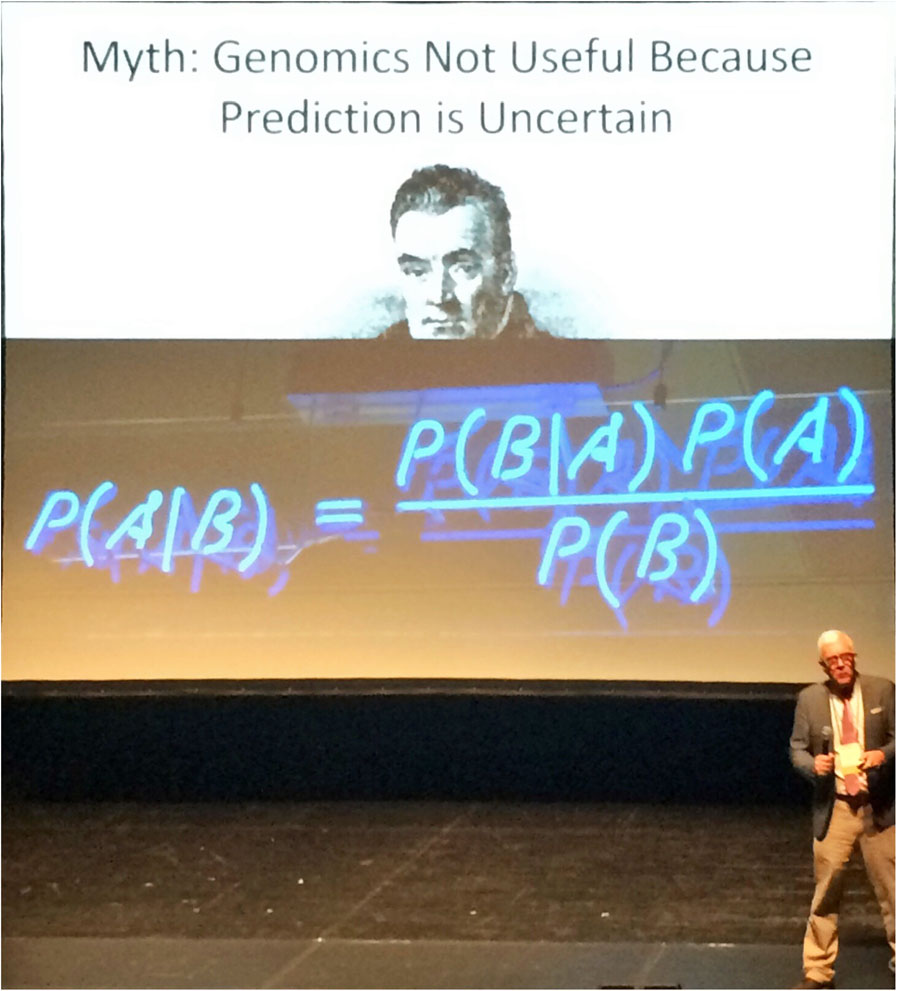
Les Biesecker giving his plenary presentation at HGM2019 in Seoul, Korea, on the future of clinical genomics

One of the highlights of the HGM in Seoul was the presentation by Leslie Biesecker (NHGRI, the National Human Genome Research Institute, USA, and current President of the ASHG, the American Society of Human Genetics; Fig. 1) entitled “The Myths of Clinical Genomics.” Les persuasively argued for an urgent change of paradigm in the use of genomics in medicine: moving away from treating a patient’s disease to preventing it in the first place. He also recommended balancing risks and benefits in medical genomics. Finally, Biesecker proposed a “positivist view” of genomics embracing widely implemented genetic testing, secondary findings, carrier screening, and pharmacogenetics. The talk led to a lively discussion with the audience including a comment by one of us (JKVR) that genomics had already once before precipitated a radical cultural change when we transitioned from genetics into genomics. This change included switching from hypothesis-driven research to more discovery-based investigations, at the time often termed “fishing expeditions.”

V. Narry Kim (Institute of Basic Sciences, South Korea) presented her lecture entitled “RNA Terminal Modification in Gene Regulation.” She described a novel technology developed in her lab called TAIL-seq which reveals the 3′-end sequences of RNA. Using TAIL-seq she discovered that certain mRNAs have mixed poly(A) tails, and deadenylation of these mRNAs is stalled in the presence of G, U, or C bases. Furthermore, her recent work has shown that TENT4A/B (which are terminal nucleotidyltransferases) produce mixed tails which shield mRNAs from rapid degradations. Narry’s work has revealed a new factor in post-transcriptional regulation. Kim also discussed the potential of using mixed tailing as an RNA-based gene therapy which could enhance the stability of mRNAs.

## Noteworthy sessions from the parallel tracks

In the microbiomics session, Eran Segal (Weizmann Inst, Israel) presented major strides in personalizing antidiabetic diets, by calibrating the stabilizing vs. destabilizing effects on blood glucose level of large numbers of nutrients in a 1000-p Israeli cohort. The effects of the nutrients and composite diets on the prediabetic state turns out to correlate with lifestyle, metabolome, microbiome, and clinical data. His group developed an algorithm which reproducibly predicts the stabilizing vs destabilizing effects of different isocaloric diets in different people. Strikingly, some meals that caused pronounced glucose peaks in one person had only mild effects on others. The resulting personalized prediabetes prevention approach is now being employed in Israel and the USA. Second, also remarkable, the microbiome of persons with different ancestry but the similar environment was found to be largely similar, suggesting that gut microbiome composition is dominated by the environment rather than by genetics. Finally, specific microbial subgenomic variations (SGV) were found, which associated with host risk factors both in the Israeli cohort and in the Dutch Lifelines cohort. In the next talk, Cisca Wijmenga (UMC Groningen, Netherlands) used the same Lifelines cohort to take gut biology and epidemiology another step further by creating 3D mini-guts-on-a-chip from hiPSC cells from the Lifelines-DEEP cohort, a section of Lifelines extensively typed by host and microbiome sequencing, genetics, metabolomics, and deep phenotyping. The chip-based system allows for controlled modeling of internal and external intestinal stimuli, including cultured microbes, that can be analyzed by imaging and cellular and molecular read-outs. Such gut-on-a-chip systems have great potential to improve our understanding of multifactorial diseases like inflammatory bowel disease and celiac disease and may help to accelerate the development of therapies. In the last talk of this session, George Weinstock (Jackson Lab, USA) showed the utility of well-designed inbred and crossbred mouse strains to study the host genetic control of microbial communities.

In the cancer genomics session, Nick Papadopoulos (Johns Hopkins, USA) presented the design and progress of the CancerSEEK approach, using multi-omics based on DNA variants in oncogenes and proteomics of a judicious selection of oncoproteins to detect subclinical, undiagnosed cancers as well as determining the cancer type. This approach, based on large datasets of UK Biobank, is expected to greatly improve the success of the early intervention. Currently, the work is in the validation stage using prospective samples from several cohort studies. In the next talk, Nic Waddell (Berghofer Institute, Australia) gave an overview of the current status and progress of NGS-based immunotherapy, highlighting the search for individualized predictors of the success or failure of the current modalities of cancer immunotherapy.

The single-molecule session contained some outstanding technological highlights of the meeting. In the first talk, Long Cai (CalTech, USA) blew away the audience by identifying, locating, and quantitating up to 10,000 transcripts in formalin-fixed single cells and tissue sections in situ*.* He did this by reproducibly hybridizing and washing, in up to 80 successive rounds, complex probe libraries labeled with 3 fluorescent channels. This seqFISH+ system generates unique temporal bar codes for all the RNAs in the sample, much like sequencing, thus yielding the required detection complexity. Indeed, the sequentiality is capable of resolving signals well beyond the optical diffraction limit of the microscope and cell size. Examples were shown of subcellular RNA localization, resolution of processes in interacting cells, and following the fate of nascent RNAs using intronic probes. Due to the power of the technology, it is plausible that direct in situ sequencing may even substitute the broadly used single RNA sequencing in large-scale consortia projects. In the next talk, Efrat Shema (Weizmann Institute, Israel) applied single-molecule imaging of individual nucleosomes as a tool to decipher the histone code at single nucleosome level, and combined this with sequencing the attached DNA to derive the precise genomic position of the epigenetic marking under study. This high-density fluorescent probing approach establishes multi-layered comprehensive data of the epigenome and seems amenable to a plethora of thus far intractable biological queries. In a third remarkable talk, Roser Vento (Wellcome Sanger Institute, UK) analyzed the female reproductive tissue using first-trimester placentas with matched maternal blood and decidual cells with the single-cell transcriptome. She analyzed HLA and immunomodulation, elaborating transcriptional patterns related to health and diseases, including pre-eclampsia, which is related to HLA. To get broader biological insights on how reproductive tissues work, she has constructed an interactome map of all the secreted peptides versus all receptors that are expressed in all the cells: such an approach is very useful to understand how cells are likely to interact during early human pregnancy.

In the session on consanguinity/rare genetics and carrier screening Fowzan Alkuraya (King Faisal Specialist Hospital and Research Centre, Saudi Arabia) presented his extensive experience utilizing high throughput DNA sequencing of recessive disorders in Saudi Arabia. The application of this technology has allowed the characterization of the genetic basis of different Mendelian diseases not only in research settings but also in the clinic. He elegantly demonstrated that as the data continue to accumulate, our understanding of genes, pathways, and molecular mechanisms will continue to evolve and translate into better diagnosis, prognosis, and therapies for these severe disorders prevailing in this country. Eva Maria Cutiongco-De La Paz (University of the Philippines, Manila, Philippines) presented her interesting data concerning “Identifying Genetic Variants Conferring Susceptibility to Infection in the Indigenous Population of the Philippines.” She demonstrated that common and rare FUT2 and/or A2ML1 gene variants confer susceptibility to otitis media, likely by modifying the middle ear microbiome through regulation of A antigen levels in epithelial cells. These studies suggested that multiple combinations of common and rare genetic variants plus environmental factors influence the individual otitis media phenotype as a complex trait.

An important workshop on recent aspects of Open Science organized by the HUGO Committee on Ethics, Law, and Society (CELS), the Human Variome Project (HVP) and the Ewha Institute for Biomedical Law and Ethics also took place. Many aspects were discussed. Participants paid attention to the role of open education and skills, Next Generation Sequencing metrics, the changing business models for scientific publications, the involvement of citizens science, and research integrity. It is important to develop new models that bind together “open science” and “open innovation,” experimenting with new models of investment, to offer stakeholders opportunities to accelerate innovation in this context. Some colleagues emphasized the right of citizens to benefit from science and the potential harm to health systems due to the non-use of data. At this point, the need was suggested to increase science infrastructures and the participation of citizens for more sustainable health care systems (e.g., bioinformatics, biobanks, and databases that are accessible by researchers, health care specialists, citizens).

James Keck (The Jackson Laboratory, USA) was a speaker in one of the two luncheon seminars of the last day, with the title “Utilization of New Humanized Mouse Models to Better Understand Emerging Immunomodulation Therapies.” He delivered a very challenging lecture on the utilization of the humanized mouse model in the most recent impacts in oncology and therapies based on immunomodulation. The characterization of these models to be used in immune oncology was depicted and the expectations, when more data will be obtained, about the new humanized model for natural killer cells are being obtained.

**Fig. 2:**
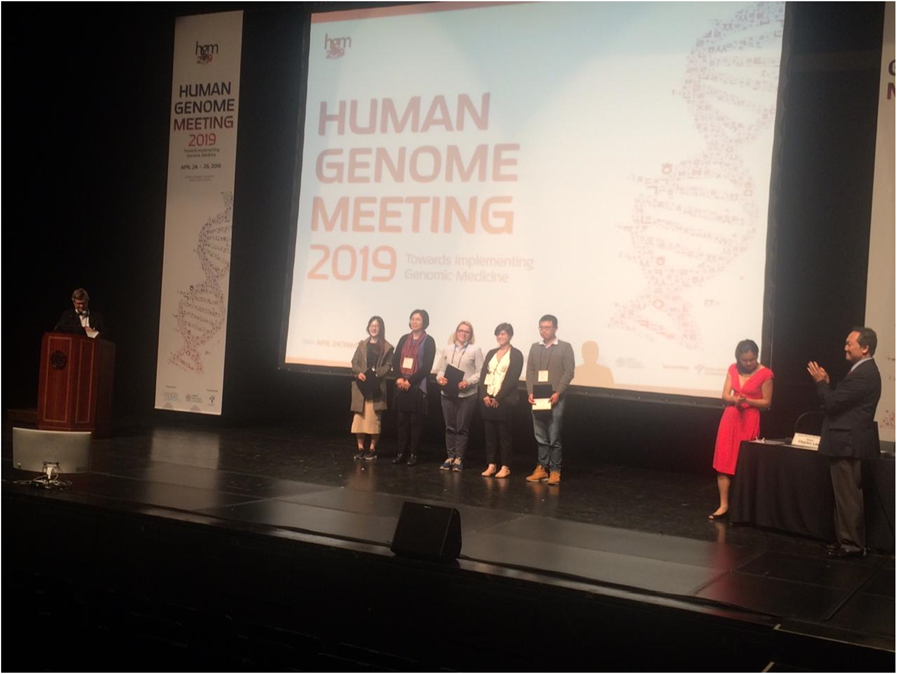
Trainee awardees honored for their contributions at the HGM in Seoul

The Human Genome Meeting, for the second time after the previous one in Yokohama, featured a special session dedicated to trainees alone, in order to provide a venue for students and postdocs to interact with each other and established scientists invited for the events. Fourteen excellent short talks were selected from the submitted abstracts, which were presented between specials talks from seasoned scientists like Cisca Wijmenga, Jonas Korlach, and David Bentley, who gave outstanding advice to trainees. Advice ranged from risk-taking (take the risk to explore new avenues!) to the importance to develop original approaches and technologies, which will always provide new types of data leading to new biological understanding. The best trainees were awarded prizes (Fig. 2). The HGM also featured the Chen Awards again. To provide a deep sense of the local culture and cuisine, the meeting featured a wonderful conference dinner featuring local entertainment.

## Conclusions

The twenty-third Human Genome Meeting brought together over 500 participants from around the world to discuss recent advances in human genomics. Scientific highlights include placing genomics at the center of human wellbeing and disease prevention, advances in cancer and single-cell genomics, vibrant investigations of the microbiome, acknowledgment of the contributions of trainees, the future of scientific progress, and much more. Finally, planned future HGMs include Perth, Australia, next year, 2020, and Tel Aviv, Israel, in 2021 highlighting the worldwide nature of the HGMs.

## Data Availability

Data sharing not applicable to this article as no datasets were generated or analyzed during the current study.

## Abbreviations

A2ML1: Alpha-2-macroglobulin-like 1
ASHG: American Society of Human Genetics
CELS: Committee on Ethics, Law, and Society
FUT2: Galactoside 2-alpha-L-fucosyltransferase 2
HGM: Human Genome Meeting
HUGO: Human Genome Organization
HVP: Human Variome Project
NHGRI: National Human Genome Research Institute
NIH: National Institutes of Health
NIPT: Non-invasive prenatal testing
SGV: Subgenomic variation

